# Supplementary material for: Mechanisms governing the pioneering and redistribution capabilities of the non-classical pioneer PU.1
Source: Nat Commun. 2020 Jan 21;11:402. doi: 10.1038/s41467-019-13960-2 (PMC6972792; doi:10.1038/s41467-019-13960-2)
Supplement: Supplementary file 7 — Source data [file 41467_2019_13960_MOESM7_ESM.zip › Source_Data/Figure6/Figure6B_MotifScanOutput/ETS1_mutPU.1/homerResults/motif5.similar.html]

motif5

## Information for motif5

T
G
C
A
A
C
G
T
A
C
T
G
G
C
T
A
C
T
A
G
A
C
G
T
T
A
G
C
C
G
T
A
A
G
C
T
  
Reverse Opposite:  

T
C
G
A
A
C
G
T
A
T
C
G
C
G
T
A
A
G
T
C
C
G
A
T
T
G
A
C
C
G
T
A
A
C
G
T
  

|  |  |
| --- | --- |
| p-value: | 1e-246 |
| log p-value: | -5.683e+02 |
| Information Content per bp: | 1.848 |
| Number of Target Sequences with motif | 1514.0 |
| Percentage of Target Sequences with motif | 11.23% |
| Number of Background Sequences with motif | 1080.4 |
| Percentage of Background Sequences with motif | 3.08% |
| Average Position of motif in Targets | 99.6 +/- 53.0bp |
| Average Position of motif in Background | 100.6 +/- 59.0bp |
| Strand Bias (log2 ratio + to - strand density) | 0.1 |
| Multiplicity (# of sites on avg that occur together) | 1.07 |
| Motif File: | file (matrix) reverse opposite |

### Similar de novo motifs found

|  |  |  |  |  |  |  |  |
| --- | --- | --- | --- | --- | --- | --- | --- |
| Rank | Match Score | Redundant Motif | P-value | log P-value | % of Targets | % of Background | Motif file |
| 1 | 0.941 | T C G A G C A T A C T G G C T A A T G C C G A T G T A C G T C A A G C T G T A C | 1e-237 | -545.819818 | 9.57% | 2.32% | motif file (matrix) |
| 2 | 0.928 | C T A G C A T G T G C A A C G T C A T G G C T A A T C G C G A T T G A C G C T A A C G T | 1e-234 | -540.712785 | 9.94% | 2.53% | motif file (matrix) |
| 3 | 0.925 | T C G A T A C G C T A G T C G A C A G T C A T G C G T A T A G C C G A T A T G C C G T A A C G T G T A C | 1e-225 | -518.176550 | 9.74% | 2.53% | motif file (matrix) |
| 4 | 0.936 | T C G A C A G T A C T G C G T A T A C G C A G T A G T C G T C A A G C T A G T C G A T C A T G C | 1e-219 | -506.488610 | 8.83% | 2.12% | motif file (matrix) |
| 5 | 0.925 | C T A G T C G A A C G T A C T G C G T A A T G C A C G T A G T C C G T A A T C G T G A C T C G A C T A G T A C G | 1e-212 | -490.012184 | 8.58% | 2.07% | motif file (matrix) |
| 6 | 0.969 | A C G T A C T G G T C A T A C G A C G T G T A C C T G A A G C T | 1e-208 | -479.928211 | 9.27% | 2.47% | motif file (matrix) |
| 7 | 0.953 | A C G T A C T G C G T A A C T G A C G T A G T C C G T A | 1e-191 | -441.277055 | 9.78% | 2.94% | motif file (matrix) |
| 8 | 0.801 | A C G T C A G T T G C A T C A G A C G T A G T C C G T A | 1e-172 | -397.287740 | 16.89% | 7.86% | motif file (matrix) |
| 9 | 0.849 | A G C T A C T G C G T A C G T A A C G T A G T C C G T A A G C T | 1e-15 | -35.387646 | 1.51% | 0.69% | motif file (matrix) |
